# Proctitis in patients with monkeypox infection: a single-center analysis of 42 consecutive cases from a multidisciplinary observational study on monkeypox proctitis

**DOI:** 10.1007/s10151-023-02782-6

**Published:** 2023-04-22

**Authors:** J. Guevara-Martínez, F. Prieto La-Noire, P. Arteaga-Asensio, I. Pascual-Miguelañez, R. Moraes Souza, M. Quiles, M. Montes, C. Fondevila, M. Álvarez-Gallego, Monkeypox multidisciplinary collaboration group, Elena Sendagorta, Natalia Gonzalez-Alcolea, Camilo Zapata-Syro, Alfredo Maldonado, Claudia Sanz González, J. L. Marijuan

**Affiliations:** 1 General and Digestive Surgery, Hospital Universitario La Paz. Universidad Autónoma de Madrid, Madrid, Spain; 2 Dermatology Department, Hospital Universitario La Paz, Madrid, Spain; 3 Microbiology Department, Hospital Universitario La Paz, Madrid, Spain; 4 Internal Medicine Department, Hospital Universitario La Paz, Madrid, Spain; 5 Instituto de Investigación, Hospital Universitario La Paz, Madrid, Spain

**Keywords:** Monkeypox, Proctitis, Outbreak, Anal abscess, Surgical drainage

## Abstract

**Background:**

The current monkeypox (MP) virus outbreak was declared an international emergency in July 2022. The aim of this report is to describe our initial experience with patients with MP, focusing on proctitis.

**Methods:**

We conducted an observational study between 20 May and 31 July 2022, on patients with MP at a reference tertiary center in Madrid, Spain. A descriptive analysis on MP was performed, focusing on its characteristics, symptoms, diagnosis, and outcomes.

**Results:**

A total of 143 positive MP cases were diagnosed in our center; 42 of them [all male, median age 39 years (range: 22–57 years)] had proctitis (29.37%), and 3 patients (2.09%/MP total cases and 7.14%/MP proctitis) required surgical drainage of a perianal abscess.

**Conclusions:**

General and digestive surgeons must be aware of the presence of proctological impairment and complications due to MP virus.

## Introduction

Monkeypox (MP) virus was first described in 1958 as an orthopoxvirus in the *Poxviridae* family. It is considered an endemic disease in Central and West Africa [1], but since May 2022, patients diagnosed with MP have been reported to the World Health Organization (WHO) from 12 member states. The eradication of smallpox justifies the low percentage of the vaccinated population and is hypothesized to play a role in the emerging outbreak [1, 2].

Currently the most important transmission route is direct contact with skin lesions, with an overall mortality rate of less than 3% [1, 3]. The vast majority of cases are among men who have sex with men (MSM), and sexual contact is thought to be the possible transmission route in up to 95–99% of cases in some series [4–6]. After direct contact from mucosal or skin lesions, the virus replicates in the inoculation site and may migrate into regional lymph nodes [7]. Following this initial period, the virus spreads into other organs with an incubation period of 7–14 days. A second viremia occurs within 1–2 days with mild symptoms that may be unnoticeable or include fever, myalgia, asthenia, and lymphadenopathy. This second viremia is associated with papules or pseudopustules with a necrotic center (mostly oropharyngeal or anogenital), and in some cases this rash may spread in a centripetal manner [5, 7]. These skin lesions persist over a 2–4-week period and synchronously evolve through macular, papular, vesicular, and pustular phases. After the pustular phase, a crust covers the lesions and desquamates in 7–14 days. Symptoms typically resolve in 3–4 weeks, and patients are considered no longer infectious after the crusts are completely healed (7–9).

On 23 July 2022, the WHO declared MP an international emergency and few articles in the literature describe the anal or rectal characteristics of this disease. The aim of this study was to describe our initial experience focusing on rectal impairment in patients with MP.

## Materials and methods

 Our study was conducted on all patients diagnosed with MP between 20 May and 31 July 2022 at the Hospital Universitario La Paz, a tertiary referral center in Madrid. The Dermatology or Internal Medicine Departments initially assessed all possible MP cases. Patients were included in compliance with the ethical committee regulations of the Hospital Universitario La Paz (Internal 2022.185, HULP: PI 5296), providing informed consent to include their data in the study.

### Diagnosis

When compatible symptoms, epidemiological background (sex with a new or multiple partners over the last weeks), and typical skin lesions were observed, swabs of skin blisters or rectal or oral ulcers, depending on symptoms, were obtained for real-time polymerase chain reaction (PCR) diagnosis of MP during the initial physical exam in the emergency department. Samples were analyzed by the Microbiology Department. All genital ulcers were analyzed for MP virus and sexually transmitted diseases (STDs), including *H*. *ducreyi*, syphilis, venereal lymphogranuloma, and herpes simplex virus (HSV) I and II.

Proctitis is defined as an inflammatory syndrome of the anal canal and/or the rectum and its common symptoms include proctalgia, tenesmus, bleeding, or mucopurulent discharge [9]. In our study, those patients with proctitis symptoms and positive rectal MP PCR results were considered to have MP proctitis. Rectal PCR samples were obtained only from those patients who complained of rectal symptoms and analysis also included STDs screening (*C*. *trachomatis*, lymphogranuloma, *M*. *genitalium*, *N*. *gonorrhoeae*, *T*. *vaginalis*, and HSV). When skin lesions were the only MP symptom without rectal symptoms, we did not perform PCR of a rectal swab. Rectoscopy was not routinely performed due to its poor tolerance and inconclusive findings.

### Management

The main goal of MP proctitis treatment is pain control. Paracetamol is usually helpful, but in cases of intractable pain, nonsteroidal antiinflammatory drugs (NSAIDs), tramadol, or viscous 2% lidocaine has been used with good results. Treatment with ceftriaxone and doxycycline was initiated according to previous guidelines for STDs until PCR results were available. Revaluation was recommended if there was persistent discomfort or worsening of symptoms. The same follow-up regime was established for well/controlled human immunodeficiency virus (HIV) infection.

In cases of persistent or worsening proctitis symptoms, the General Surgery Department was contacted. When surgical drainage was needed, the surgical technique, anesthesia, and postoperative management was similar to the usual cryptoglandular abscess. FFP-2 masks, surgical gowns and goggles, or face shields are recommended to avoid droplet contact. During hospital admission, rigorous infection control procedures were followed.

## Results

A total of 208 patients were tested for MP, with a total of 143 positive cases (68.75%), and 42 patients were diagnosed with MP proctitis (29.37%).

All patients with proctitis were male, with a median age of 39 years (range: 22–57 years). In total, 31 patients (74.35%) stated having anal sex without protection or with a new partner in the previous days, with a median duration of rectal symptoms before MP diagnosis of 5 days (range 1–25 days). Patients who stated having previous anal sex were tested for STDs; PCR of a rectal swab was positive in four patients (9.52%) as presented in Table 1. In five patients, PCR of a swab of skin lesions was also positive for STDs, including syphilis and HSX 1 and 2, and 27 patients had a well-controlled HIV infection (64.28%).

**Table 1:** Demographics and clinical data of patients with MP proctitis

Patient	Age (years)	Anal receptive sex	Positive rectal swab PCR	Positive skin swab PCR	Anogenital skin lesions	HIV	STD Skin	STD Rectal	Antiviral treatment	Days with proctitis symptoms before diagnosis	Tenesmus	Diarrhea	Anal abscess	Emergency surgery	Admission	Other mucosal lesions
1	40	Yes	Yes	Yes	Yes	Yes	Syphilis	No	Yes	2	Yes	Yes	No	No	No	Pharynx
2	39	Yes	Yes	No	No	Yes	No	No	Yes	7	Yes	No	No	No	No	No
3	35	Yes	No	Yes	Yes	No	No	No	No	21	Yes	Yes	No	No	No	No
4	39	Yes	Yes	Yes	Yes	Yes	No	No	No	5	Yes	No	No	No	No	No
5	49	Yes	Yes	Yes	Yes	No	No	No	No	6	No	No	No	No	No	No
6	22	No	Yes	No	Yes	No	No	No	No	5	No	No	No	No	No	No
7	51	Yes	Yes	Yes	Yes	Yes	No	No	No	4	No	No	No	No	No	No
8	20	Yes	Yes	Yes	Yes	No	No	No	No	4	Yes	Yes	No	No	No	Face
9	39	Yes	Yes	No	No	Yes	No	No	Yes	19	No	No	No	No	No	Oral
10	50	Yes	Yes	No	No	Yes	No	No	No	5	No	No	No	No	No	No
11	43	Yes	Yes	No	No	Yes	No	No	No	1	Yes	Yes	**Yes**	Yes	Yes	No
12	26	No	Yes	No	No	No	No	No	No	1	No	No	No	No	No	No
13	39	Yes	Yes	Yes	Yes	Yes	HSV 1	HSV 1	No	1	Yes	Yes	No	No	No	No
14	29	No	Yes	No	Yes	Yes	HSV 2	No	No	3	Yes	No	No	No	No	Pharynx
15	46	No	Yes	No	No	Yes	No	No	No	7	Yes	No	No	No	No	Pharynx
16	44	Yes	Yes	No	No	Yes	No	No	No	6	Yes	No	No	No	No	No
17	42	No	Yes	No	Yes	Yes	No	No	No	7	No	No	No	No	No	No
18	35	Yes	Yes	No	No	Yes	No	No	No	7	Yes	No	No	No	No	No
19	37	No	Yes	Yes	Yes	Yes	No	HSV 2 and *N*. *gonorrhoeae*	No	2	Yes	No	No	No	No	No
20	45	Yes	Yes	Yes	Yes	Yes	No	No	No	4	Yes	No	No	No	No	No
21	49	No	Yes	No	Yes	Yes	No	No	Yes	1	No	No	No	No	No	No
22	28	Yes	No	Yes	Yes	No	No	No	Yes	25	No	No	**Yes**	Yes	Yes	No
23	30	No	Yes	No	No	No	No	No	No	4	No	Yes	No	No	No	Pharynx
24	27	Yes	Yes	No	Yes	Yes	No	No	Yes	7	Yes	No	No	No	No	Pharynx
25	62	No	Yes	No	No	Yes	No	No	No	10	Yes	Yes	No	No	No	Oral
26	47	Yes	Yes	Yes	Yes	Yes	No	No	Yes	5	No	No	No	No	No	No
27	38	Yes	Yes	No	Yes	Yes	No	No	Yes	13	No	No	No	No	No	No
28	34	Yes	Yes	Yes	No	No	No	No	Yes	9	No	No	**Yes**	Yes	Yes	No
29	26	Yes	Yes	No	No	No	No	No	No	3	No	No	No	No	No	No
30	32	Yes	Yes	Yes	Yes	Yes	SHV1	No	No	2	Yes	No	No	No	No	No
31	34	Yes	Yes	No	Yes	No	No	No	Yes	14	Yes	Yes	No	No	No	No
32	40	Yes	Yes	No	No	Yes	No	No	No	5	Yes	Yes	No	No	No	Pharynx
33	57	Yes	Yes	No	Yes	Yes	No	No	No	5	Yes	No	No	No	No	No
34	22	Yes	Yes	No	No	Yes	HSV 1	*N*. *gonorrhoeae*	Yes	4	Yes	Yes	No	No	No	Oral
35	44	Yes	Yes	No	No	No	No	No	No	2	Yes	Yes	No	No	Yes	No
36	44	Yes	Yes	No	No	No	No	*C*. *trachomatis*	No	4	Yes	Yes	No	No	No	No
37	33	Yes	Yes	No	No	No	No	No	No	4	Yes	Yes	No	No	No	No
38	32	Yes	Yes	No	No	Yes	No	No	Yes	11	Yes	Yes	No	No	No	Oral
39	53	Yes	Yes	No	Yes	Yes	No	No	Yes	8	Yes	No	No	No	No	Oral
40	40	Yes	Yes	No	No	No	No	No	No	11	Yes	No	No	No	No	Oral
41	34	No	Yes	No	No	Yes	No	No	Yes	10	No	No	No	No	No	No
42	41	No	Yes	No	No	No	No	No	No	7	No	No	No	No	No	No

*MP* monkeypox, *PCR*   polymerase chain reaction; *HIV*   human immunodeficiency virus

**Fig. 1:**
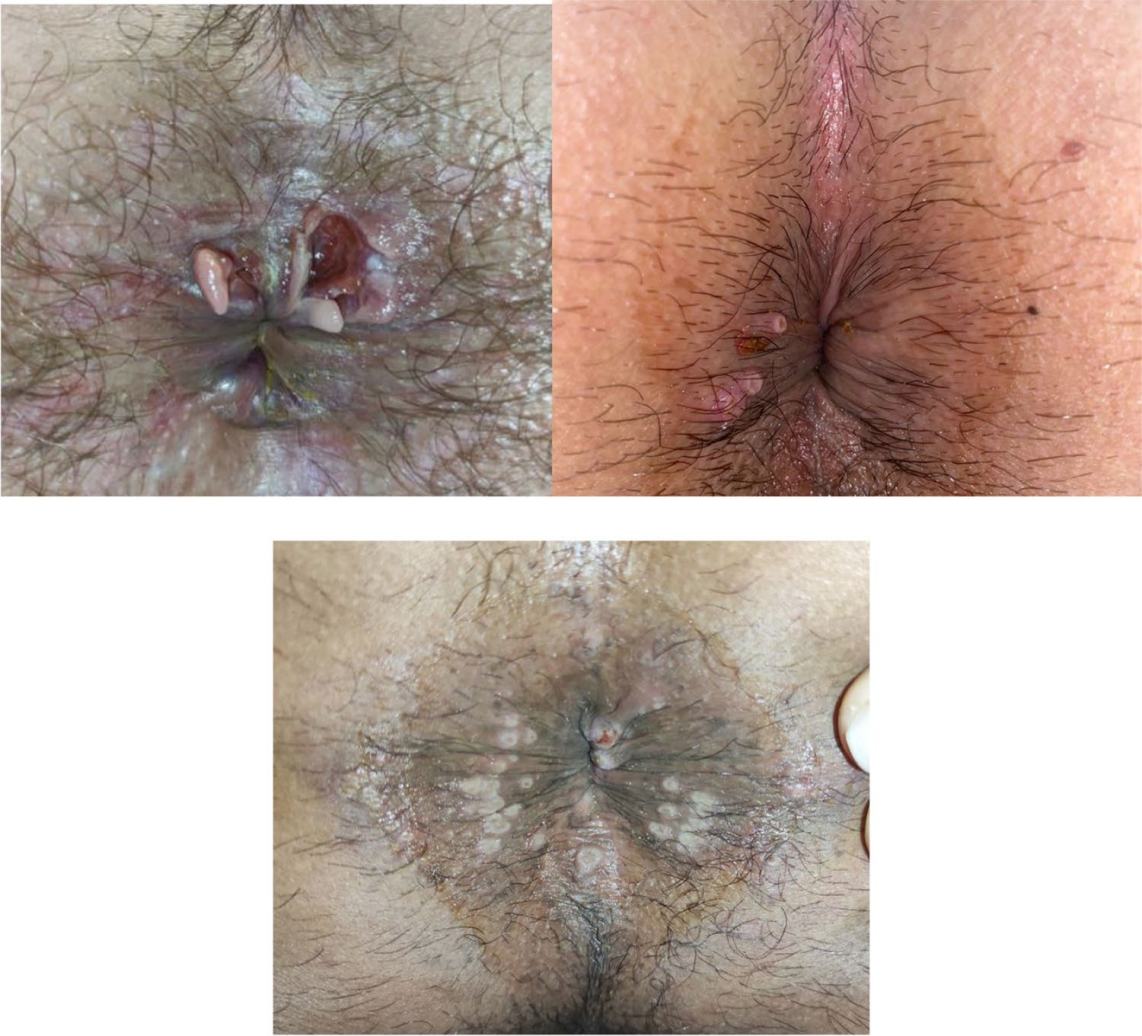
Anal lesions

A total of 21 patients with proctitis had anogenital papules or ulcers (50%) and 9 (21.42%) had only cutaneous lesions. The most common locations for cutaneous lesions apart from the anogenital region were the mouth, back, hands, and feet (Table 1). These lesions were usually seen in contact areas as papules or pseudopustules that later become umbilicated and necrotic (Fig. 1). Oral or pharyngeal infections were also observed in 12 patients with proctitis (28.57%), and typically presented as chancriform lesions.

All patients with proctitis symptoms had rectal swab PCR testing except for two (4.76%). These two patients were also the ones with the longest duration of proctitis symptoms before MP diagnosis, as presented in Table 1.

The first patient (patient 3 in Table 1) had perianal ulcers, and a swab for PCR was taken from this lesion. He reported anal discharge and proctalgia after a receptive rectal sex episode 21 days prior. His outcome was uneventful, with only paracetamol, and he did not require further medical assistance.

The second patient (patient 22 in Table 1) was diagnosed with MP 25 days prior, with almost complete resolution of cutaneous ulcers, but he presented with progressive proctalgia. During surgery, a possible external fistulous orifice was observed without an internal orifice. The postoperative course was uneventful and he was discharged 24 h after surgical drainage.

**Fig. 2:**
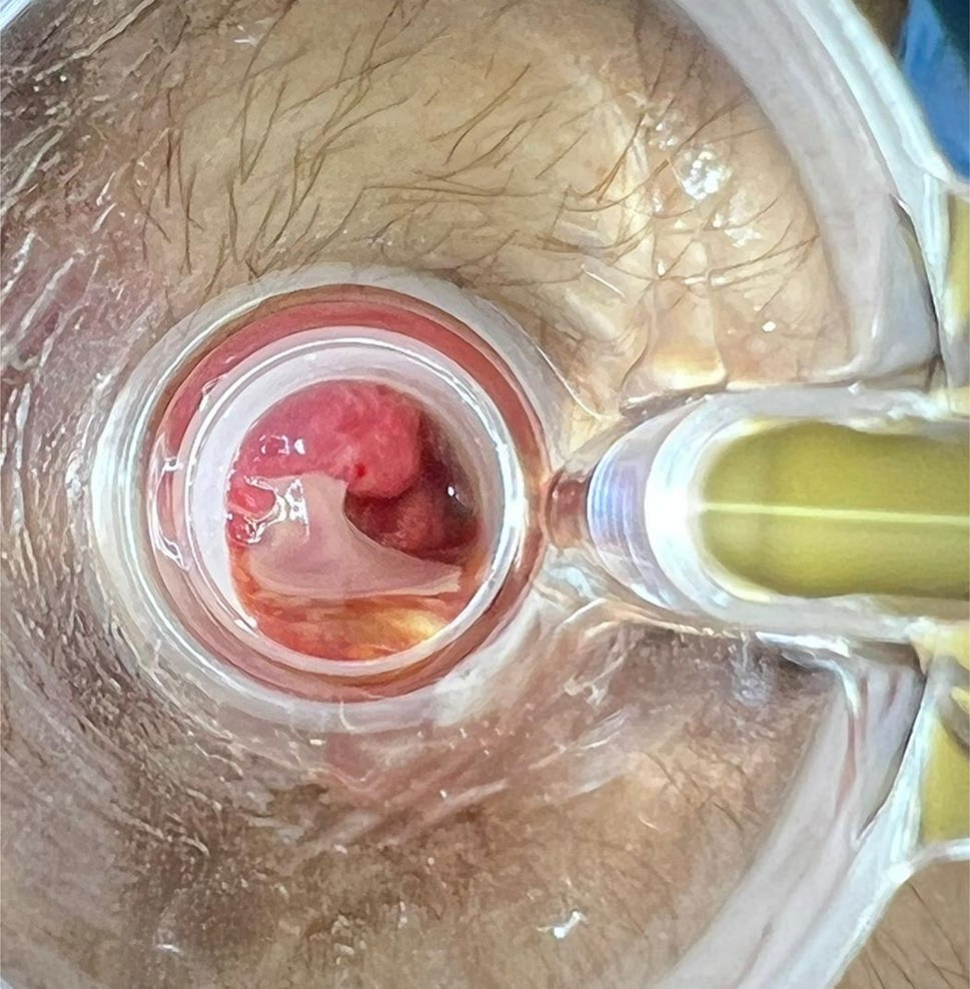
Anoscopy; anal abscess (patient 28)

**Fig. 3:**
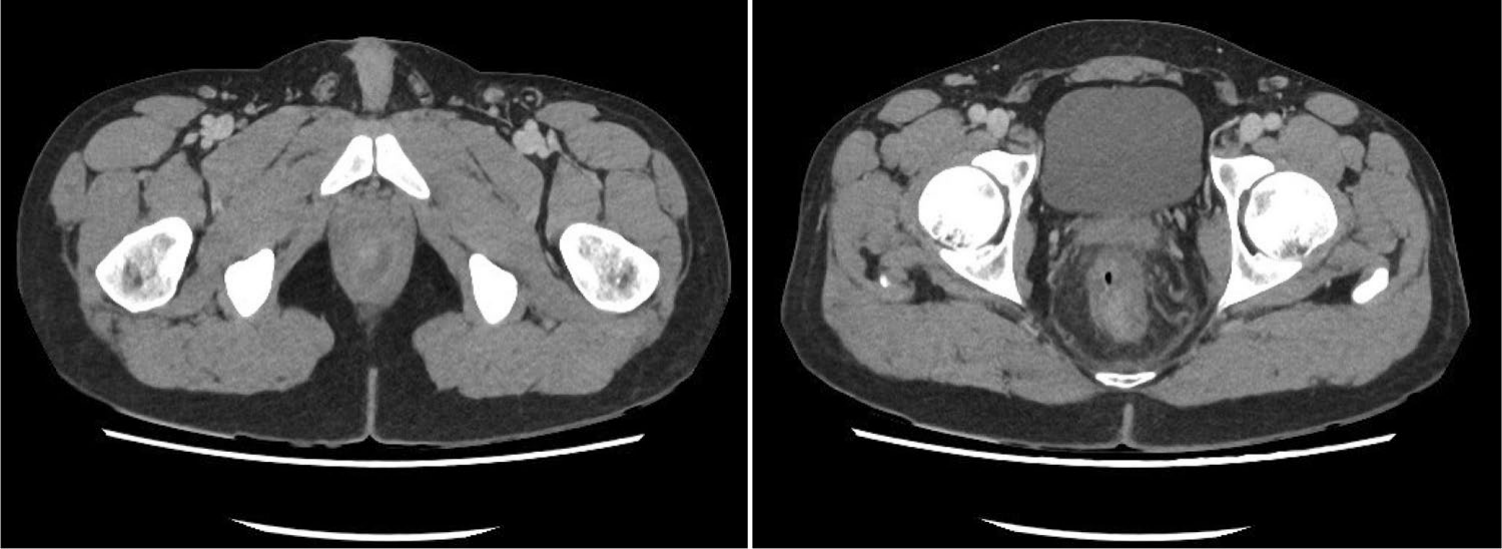
Computed tomography scan. Left lateral abscess and proctitis (patient 28)

Surgical drainage of an anal abscess was necessary in only three patients (7.14% from the proctitis group and 2.09% from the total patients with MP). All three patients were male, with a median age of 39 years (range 28–43 years), had been engaged in receptive anal sex, and were not vaccinated against MP. The first patient (patient 11 from Table 1) was also positive for rectal chlamydia when he was diagnosed with MP. The third patient (patient 28 from Table 1) had a previous diagnosis of well-controlled HIV and was referred to the emergency department by the dermatologists due to intense proctalgia; anal ulcers and mucopurulent discharge were observed during physical examination (Fig. 2). An intersphincteric abscess was suspected on digital rectal examination, and axial computed tomography (CT) scan confirmed these findings before surgical drainage (Fig. 3). In all cases, intraoperative rectoscopy evidenced intense mucosal edema and mucopurulent discharge. Patients were discharged after surgical drainage on postoperative day 1 or 2 without any complications. Oral antibiotics were not indicated upon discharge.

## Discussion

During the study period, the World Health Organization (WHO) declared an international emergency (23 July 2022) due to a rapid increase in the number of newly reported cases of MP. By August 2022, a total of 11,536 cases of MP had been diagnosed in Europe. Madrid was one of the most affected regions, with 1817 of the total of 4942 confirmed cases of MP reported in Spain by 1 August 2022.

This rapid increase in the number of newly reported cases over a short time period intensified public awareness and prompted the implementation of preventive measures, including vaccination for vulnerable populations. These measures likely played a role in slowing the spread of the MP outbreak. As a result, there was a progressive decrease in the number of MP cases, with a total of 7498 cases reported in Spain by 27 December. There have been no new cases reported in our center since October 2022.

Up to 95% of patients with MP are men [5, 6, 11]. While many of these men identify as MSM, MP transmission is not exclusive to this group and has also been reported in women and in men who have sex with women [12]. All patients with MP proctitis in our study reported practicing receptive anal sex prior to diagnosis and typically had genital or anal lesions that expand centrifugally [5, 7, 13].

In all suspected cases of MP, PCR testing of both skin lesions and rectal samples (when proctitis symptoms are present) is necessary for confirmation of the diagnosis. Upon initial contact with possible cases, healthcare workers should take precautions to prevent direct contact and transmission through respiratory droplets, such as wearing surgical masks and gloves [2, 9, 14–16]. A study by Nörz et al. found viral deoxyribonucleic acid (DNA) on surfaces in hospital rooms, as well as on linen and personal protective equipment (PPE) worn by healthcare workers after contact with an patient positive for MP. While the presence of the virus on these surfaces may not always lead to transmission, direct contact with mucosa, damaged skin, or high virus loads could result in the spread of the infection [16].

Proctitis has been reported in approximately 22–25% of patients with MP in previous studies, with common symptoms being rectal mucosal swelling and a mucopurulent discharge [5, 17]. A majority of patients also experience painful skin ulcers or papules around the anus [18]. In cases of proctitis where the patient has a history of receptive anal sex, it is important to test for and exclude MP and other STDs, as these infections may occur simultaneously and should be treated together. Other anorectal pathogens that can cause proctitis include *Neisseria gonorrhoeae*, *Chlamydia trachomatis*, *Treponema pallidum*, and herpes simplex virus, which are among the most common causes [8]. STDs such as chlamydia, lymphogranuloma venereum, and syphilis are typically associated with rectal symptoms including mucosal swelling, discharge that is mucopurulent and may be bloody, and ulcers in cases of infection with the latter two pathogens [8, 11]. These symptoms are similar to what was observed in MP proctitis cases without any additional STDs. Therefore, rectoscopy is not considered mandatory for diagnosis but it may provide useful information in cases of severe pain, and a general surgeon should be consulted to rule out the presence of an abscess.

One limitation of our study is that a patient who required surgery was not tested for MP or other STDs with rectal swab PCR and had rectal symptoms for 25 days after a diagnosis of MP was made, so abscess formation cannot be solely attributed to the presence of MP or other STDs. The severe swelling of the rectal mucosal and discharge seen during rectoscopy ruled out a typical cryptoglandular abscess. Another limitation was that we only obtained rectal PCR from those patients with proctitis and not from all MP cases; therefore, we cannot confirm that rectal samples are negative for virus detection in those patients without rectal symptoms. This mechanism could play a role in its transmission during early stages of the disease or in those patients with mild symptoms. PCR was always obtained from rectal/oral mucosa or skin blisters, but we cannot confirm that there were patients with mild skin lesions who required surgical drainage without being tested nor suspected for MP infection. Furthermore, all patients with proctitis are routinely tested for STDs (and MP virus since the beginning of the outbreak) during the first contact as previously described; therefore, it is unlikely that we missed proctitis cases without abscess, considering the range of days with rectal symptoms (range 1–25 days) before MP diagnosis.

Due to the severe rectal mucosa edema, physical exploration may wrongly suggest the presence of a rectal abscess. If rectal findings are inconclusive, CT scan is recommended. Severe circumferential anorectal mural thickening with hypoattenuated zones is the most common radiological finding, indicating mucosal edema and rectal ulcers [19]. As in the previously described rectoscopy findings, these must be accompanied by a compatible epidemiological context, typical skin lesions, and PCR study for diagnosis, since they are typical of proctitis due to either MP, STDs, or other causes such as radiation or ischemia [19].

If an abscess is diagnosed, surgical drainage should be made with the donning of full personal protective equipment to avoid contact with droplets [14, 15]. The surgical drainage and postoperative follow-up by the general surgeon is similar to treatment for a cryptoglandular abscess, with a consultation around 1 month after surgery. Internal medicine and dermatology departments continue follow-up, as was the case for all the patients with MP diagnosed in our center. Our patients were advised to seek medical assistance in our center if their condition worsened. Many patients had direct medical access through their HIV consultation, which may permit early diagnosis and limit disease spread, although it has been reported that patients with well-controlled HIV infections with an undetectable viral load and a high CD4 count are less likely to have a severe course [20]. In our study, we did not prescribe oral antibiotics after discharge and we did not have any readmissions or reoperations.

In observational studies, a cross-immunity for monkeypox infection using the smallpox vaccine has been described by the WHO, although most of the population under 40–50 years of age probably will not benefit from the protection provided by this vaccine applied during childhood [21]. On 12 July 2022, the Spanish Health Council recommended the administration of a single dose of IMVANEX in pre-exposure, high-risk populations (including patients with HIV with follow-up who are not previously vaccinated nor infected by smallpox). A dose of vaccine in the first 4 days post-exposure is recommended for immunocompromised patients, pregnant women, and children. This vaccination is also recommended for health personnel with direct MP exposure with patients or samples, and for direct contact with positive cases [22, 23]. Since our cohort was diagnosed and treated between 20 and 31 May 2022, vaccination was not generalized, and its protective effect against MP proctitis cannot be determined since we did not have any vaccinated patients in our proctitis cohort. A drastic decrease in the number of new MP cases may be attributed to the implementation of a vaccination campaign by the Health Ministry.

The current treatments for anal and rectal lesions include NSAIDs, prevention, and surveillance. Antivirals such as the VP37 assembly protein inhibitor tecovirimat have been registered by the European Medicine Agency for treating MP and cowpox since January 2022 [1, 2]. The oral drug brincidofovir (oral route) and its intravenous analog cidofovir inhibit the DNA polymerase [24, 25]. Both drugs have been shown to be effective against orthopoxvirus infections *in vitro* and in animal models, but with considerable toxicity [9]. Cidofovir may be most effective when administered early after MP exposure but it is also useful in diminishing MP manifestations in later stages of the infection.

## Conclusions

MP infection has turned out to be a re-emergent contagious disease, most common in MSM. In patients with a compatible epidemiological background and skin lesions, early protective measures should be taken to prevent the spread of the disease. PCR of samples from skin lesions and rectal samples in cases of proctitis is mandatory for diagnosis and should also include STD screening when necessary. The main focus of MP management is pain control, and early reevaluation is advised in case of deterioration. Persistent or progressively worse proctalgia indicates the presence of a perianal abscess.

## Data Availability

Data is available upon request.
